# Molecular phylogenetic analysis and morphological reassessments of thief ants identify a new potential case of biological invasions

**DOI:** 10.1038/s41598-020-69029-4

**Published:** 2020-07-21

**Authors:** Mostafa R. Sharaf, Dietrich Gotzek, Benoit Guénard, Brian L. Fisher, Abdulrahman S. Aldawood, Hathal M. Al Dhafer, Amr A. Mohamed

**Affiliations:** 1 Department of Plant Protection, College of Food and Agriculture Sciences, King Saud University, Riyadh, Kingdom of Saudi Arabia; 2 Department of Entomology, National Museum of Natural History, Smithsonian Institution, Washington, DC 20560 USA; 3 School of Biological Sciences, The University of Hong Kong, Hong Kong SAR, China; 4 California Academy of Sciences, San Francisco, CA 94118 USA; 5 Department of Entomology, Faculty of Science, Cairo University, PO Box 12613, Giza, Egypt

**Keywords:** Zoology, Entomology

## Abstract

Species delimitation offered by DNA-based approaches can provide important insights into the natural history and diversity of species, but the cogency of such processes is limited without multigene phylogenies. Recent attempts to barcode various Solenopsidini ant taxa (Hymenoptera: Formicidae: Myrmicinae), including the thief ant *Solenopsis saudiensis* Sharaf & Aldawood, 2011 described from the Kingdom of Saudi Arabia (KSA), were precipitated by the unexpected existence of a closely related species, the Nearctic *S*. *abdita* Thompson, 1989 within the *S. molesta* species complex native to Florida. This finding left the species status of the former uncertain. Here, we investigated the taxonomy and phylogeny of these two species to determine whether or not *S*. *abdita* represents a new global tramp species. We inferred a phylogeny of the two species using DNA sequence data from four nuclear genes (*Abd-A*, *EF1α-F1*, *EF1α-F2*, and *Wingless*) and one mitochondrial gene (*COI*) sampled from populations in Florida, Guatemala, Hawaii, and Saudi Arabia. Both species clustered into one distinct and robust clade. The taxonomy of *S*. *saudiensis* was re‐examined using morphometrics. A reassessment of the morphological characters used to diagnose the worker and queen castes were consistent with molecular evidence. Based on combined morphological and molecular evidences *S*. *saudiensis* is declared as a junior synonym of *S*. *abdita* (syn. nov.). In addition, our findings indicate that *S*. *abdita* is a novel global tramp species which has a far wider distribution than previously thought and has established itself in many new habitats and different geographic realms.

## Introduction

Ants are a highly adaptive eusocial arthropod group with impressive diversity and abundance and are encountered in most terrestrial ecosystems^1,2^. The cosmopolitan genus *Solenopsis* Westwood, 1840 (Formicidae: Myrmicinae) is composed of two subgroups, fire ants and thief ants. While fire ants are infamous for being aggressive and highly invasive (e.g. *Solenopsis invicta* and *S. geminata*), the majority of *Solenopsis* species belong to the thief ants, a group consisting mainly of minute, subterranean species with monomorphic or mildly polymorphic workers^3^. The genus is speciose, with 196 recognized valid species and 22 subspecies^4^ widespread in the tropics and warm temperate regions^5–7^, with a majority of species reported from the Neotropical realm^8^. Several traits, such as nest type, tramp behavior, omnivory, and social polymorphism (monogyny and polygyny in a single species), facilitate their establishment in newly colonized environments^9,10^. The fire ants of the *S. geminata* and *saevissima* species groups^11^, e.g. *S. geminata* and *S. invicta*^9^, are notorious pests. Among the most damaging invasive ants in the world^12^, they have spread around the world via human commerce^13,14^. While the expansion of urban ecosystems together with the extraordinary growth in international trade drive the spread and establishment of many species outside their native ranges^15,16^, *Solenopsis* appears particularly well-adapted to urban habitats due to their generalized diet^17^.

Biological invasions are the indirect outcome of human-mediated drivers of global change. Today, such invasions pose major challenges to agriculture and ecological balance^18^. The number of invasive species has continued to rise owing to growth in international trade and globalization^19,20^. Immediate and effective control and management strategies are predicated on accurately identifying invading pest species, placing taxonomy and systematics research at the forefront of invasive species exploration^21^. However, species identifications are not always easy since many alien insects are morphologically difficult to distinguish from native species. The absence of diagnostic morphological characters, a lack of modern taxonomic revisions and keys, poor taxonomic histories, and unknown species origin can all hamper the identification of invasive species. Morphological data^22,23^, molecular data ^24,25^, or a combination thereof^26,27^ are needed to enable the correct identification of new invasive species.

Recent research highlighted a potentially diverse fauna of *Solenopsis* on the Arabian Peninsula^28,29^ with only a single introduced species, *S*. *geminata* (Fabricius,  1804), known from the United Arab Emirates (UAE)^30,31^. Six *Solenopsis* species have been recorded from the Arabian Peninsula: *S*. *elhawagryi* Sharaf and Aldawood, 2012, *S*. *geminata* (Fabricius, 1804), *S*. *omana* Collingwood and Agosti, 1996, *S*. *saudiensis* Sharaf and Aldawood, 2011 (herein treated as a junior synonym of *S*. *abdita*), *S*. *sumara* Collingwood and Agosti, 1996, and *S*. *zingibara* Collingwood and Agosti, 1996^28–30,32^. However, only two species were recorded from the KSA, *S*. *elhawagryi* and *S*. *saudiensis*. It is likely that more species (both native and exotic) will be documented in the future, given the vast areas of the KSA that remain to be explored.

Despite the abundance and ecological significance of some fire ant species, the genus as a whole remains poorly studied. The systematics of this group has been plagued by difficulties in distinguishing species and their relationships. The group presents a paucity of constant and reliable diagnostic morphological characters coupled with evidently common intraspecific variations that go beyond interspecific differences^3,33^. These difficulties are particularly daunting in the large, polymorphic fire ant group, where the worker caste can provide useful characters for species identification^33^, but even more dire in the thief ants, which are mainly monomorphic (e.g. *S*. *saudiensis*^29^), and offer even fewer diagnostic characters for species delimitation. This surely represents a major impediment for faunistic inventories and biogeographical studies.

Members of the genus can be recognized by the following character states: masticatory margin of mandibles armed with three or four teeth; palp formula 2,2 or 1,2; clypeus longitudinally bicarinate, with a median area distinctly elevated and deeply inserted posteriorly between the frontal lobes; anterior margin of clypeus with a single long median seta; antennae 10-segmented with a two-segmented club; frontal carinae and antennal scrobes absent; propodeum unarmed^34^. However, the α-taxonomy of *Solenopsis* is still confused and identification to a species level is substantially challenging. Regardless of the virtually unknown ecology and cryptic habits of most species, two basic issues may explain the difficulty of identifying specimens of *Solenopsis*. First, worker caste morphology lacks diagnostic characters, especially for monomorphic species (e.g. *S*. *abdita*) that present troublesome intraspecific variation in morphological traits^3,33,35^. Second, most species were inadequately described due to limited material^36^. Third, rampant misidentifications, and the tendency to lump difficult-to-identify specimens into “wastebin species” groups causes bias and distorts the possible species lists of *Solenopsis* fauna for any given area. Finally, the use of numerous trinomials and quadrinomials has caused serious taxonomical ambiguities^37^.

Such taxonomic complexities have fueled a growing interest in the adoption of DNA-based approaches for ant descriptions and identifications. Character-based DNA barcoding, using short mitochondrial DNA fragments of the *cytochrome c oxidase I* (*COI*) gene, was introduced as a tool for rapid species identification or delimitation in ant surveys^38–42^ and systematic revisions^43,44^. Additionally, it represents a useful tool to assign different castes to a species. This is particularly useful where morphological differences between workers and sexuals may be insurmountable and only co-occurrence in nests or molecular methods allow robust assignment^43^. Since its introduction^45,46^, DNA barcoding has been extensively used^47–49^ and significantly refined^50–52^, but several pitfalls of barcoding approaches remain^53^. Therefore, species hypotheses based on DNA barcodes should ideally be additionally supported by additional molecular, morphological, geographical, ecological and/ or ethological data^54^.

A recent barcoding study of Saudi Arabian *S. saudiensis* unfortunately fell prone to such limitations^55^. Briefly, the authors used biased taxonomic sampling, heavy reliance on the Barcode of Life DataSystems (BOLD) data lacking solid taxonomic identifications, and inappropriate interpretation of analyses to arrive at misleading conclusions. Rasool et al.^55^ interpreted the finding of a single COI haplotype as proof that all tested *S. saudiensis* populations constituted a single and strong gene pool adapted to a specific habitat (palm trunk nesting) that was genetically isolated by significant natural barriers. Their analysis further clustered *S. saudiensis* with other morphologically unrelated species (e.g., the Malagasy *S. mameti* and the Neotropical *S. saevissima*) and placed *S. elhawagryi* with other *Solenopsis* species from The Americas based on claims of genetic similarities.

The aims of this study are (1) to add to ongoing efforts to develop a barcode reference library of *Solenopsis* species, (2) to combine both morphological and molecular evidences to investigate the phylogenetic relationship between *S*. *saudiensis* and *S*. *abdita*, (3) to place the two Saudi Arabian species (*S. saudiensis* and *S. elhawagryi*) into a larger biogeographic context using mitochondrial and nuclear gene sequences, (4) to test the conclusions of Rasool et al.^55^, and (5) to support and verify conclusions of our molecular analyses using morphological observations.

## Material and methods

### Institutional abbreviations

BMNH: The Natural History Museum (British Museum, Natural History), London, U.K.
FMNH: The Field Museum of Natural History, Chicago, IL, U.S.A.
KSMA: King Saud University Museum of Arthropods, Plant Protection Department, College of Food and Agriculture Sciences, King Saud University, Riyadh, Kingdom of Saudi Arabia.
NHMB: Naturhistorisches Museum, Basel, Switzerland.
NMNH: National Museum of Natural History, Smithsonian Institution, Washington, DC, U.S.A.

Throughout the work “w” is used to indicate worker, “m” male or males, and “q” queen.

### Sample collection and information

Samples physically accessible for use in our study are listed in Supplementary Table S1. We had access to 12 nominal *S. saudiensis* samples, one *S. abdita* sample, and two samples unidentified to species, but which we aligned with *S. abdita* (*S. cf. abdita*) based on morphological and molecular data. Additional material examined is listed below. In addition, we included representative samples from regions allowing us to identify the native biogeographic areas of *S. abdita*/*S. saudiensis* (i.e., New World, Afrotropics, Eurasia). Our sampling was informed by Shreve et al.^56^. We further obtained the seven *COI* sequences from Rasool et al.^55^, nuclear and *COI* sequence data from Shreve et al.^56^, and high-resolution automontage images of *S. abdita* and *S. saudiensis* from AntWeb^57^. In addition, we had access to Rasool’s voucher material for morphological examination. Voucher specimens are deposited at the KSMA and NMNH.

### Measurements and indices

Measurements and indices were performed as previously described^3,29,58^. All measurements are in millimeters.

TL: Total Length; the outstretched length of the ant from the mandibular apex to the gastral apex.
HW: Head width; the maximum width of the head behind eyes in full-face view.
HL: Head length; the maximum length of the head, excluding the mandibles.
CI : Cephalic Index (HW × 100/HL).
SL: Scape length, excluding basal neck.
SI : Scape Index (SL × 100/HW).
EL: Eye Length; the maximum diameter of the eye.
ML: Mesosoma length; the length of the mesosoma in lateral view, from the point at which the pronotum meets the cervical shield to the posterior base of the propodeal lobes or teeth.
PL: Petiole length; the maximum length measured in dorsal view, from the anterior margin to the posterior margin.
PW: Petiole width; maximum width measured in dorsal view.
PPL: Postpetiole length; maximum length measured in dorsal view.
PPW: Postpetiole width; maximum width measured in dorsal view.

### Molecular data generation

The phylogenetic relationships among our samples were inferred using molecular data from Shreve et al.^56^, who sequenced four nuclear genes (see below) and *COI* to estimate a global phylogeny of *Solenopsis*. Their data was subsampled to include representative New World species discussed by Rasool et al.^55^ as well as Old World species. In addition, we generated two new *S. saudiensis COI* barcodes from Riyadh, which were identical. Finally, all *S. saudiensis*, *S. abdita*, and *S. cf. abdita* samples used by Shreve et al.^56^ were re-extracted and sequenced in a different laboratory to prevent contamination and ensure that no samples were mixed up. Molecular methods follow Brady et al.^59^ and Moreau et al.^60^ Briefly, total genomic DNA was isolated from whole single workers with the Qiagen DNeasy Blood and Tissue kit (Qiagen Inc., Valencia, CA, USA). Only a single individual from each collection event was used to avoid subsampling colonies. DNA sequence data were generated from four nuclear protein‐coding genes (*Abdominal-A* (*Abd-A*), *elongation factor 1-alpha F1* (*EF1α-F1*), *elongation factor 1-alpha F2* (*EF1α-F2*), and *Wingless* (*Wg*)), and the mitochondrial protein‐coding gene *cytochrome c oxidase I* (*COI*). Primer sequences, PCR amplification, and Sanger sequencing protocols are given in Brady et al.^59^ and Moreau et al.^60^ We only deviated from the given protocols by adding BSA (0.08 mg/mL final concentration) to the final PCR reaction mix and using a touchdown PCR procedure to increase specificity, which started 5 °C above the published annealing temperatures and decreasing by 0.4 °C/cycle for 12 cycles. PCR amplicons were Sanger sequenced in both directions using PCR primers and the BigDye Terminator 3.1 kit on an ABI 3730xl capillary sequencer (Applied Biosystems, Carlsbad, CA, USA). Sequence traces were assembled in Geneious Prime 2020.05 (https://www.geneious.com) and deposited in GenBank (GenBank accession numbers MT550038–MT550618; see Supplementary Table S2). For comparison, Rasool et al.^55^ *COI* sequence data from *S*. *saudiensis* collected from the Riyadh region, KSA, were included (GenBank accession numbers KR916796–KR916802; see Supplementary Table S2).

### Molecular data analysis

We assembled two molecular datasets: a multilocus dataset derived from Shreve et al.^56^ consisting of four nuclear loci, and *COI*, to better place *S. saudiensis* within a global biogeographic framework. We also assembled a *COI* barcoding dataset to compare against the *S. saudiensis* haploytype described by Rasool et al^55^.

Each locus was globally aligned using the global iterative refinement method (G-INSI-i) implemented in MAFFT 7.402 (Katoh and Standley^61,62^;—globalpair—maxiterate 1000—retree 100). The concatenated multilocus dataset produced a 2,546 bp alignment, of which 457 nucleotides were variable and 338 were parsimony informative. Use of other alignment algorithms did not impact phylogenetic tree estimation. For each dataset, we estimated maximum likelihood trees using IQTREE 1.6.12^63^, simultaneously estimating the optimal model of nucleotide substitution using ModelFinder^64^ (multilocus: SYM + R3, *COI*: TIM2 + F + I + G4) on an unpartitioned dataset. We estimated branch support using ultrafast bootstraps^65,66^ (1,000 replicates) and two approximate likelihood-based measures (aLRT) (Shimodaira—Hasegawa—aLRT [SH-aLRT] with 1,000 replicates and the Bayesian-like transformation aLRT [aBayes]^67^. Bayesian trees were estimated using MrBayes 3.2.65^68^. For the *COI* dataset, we applied the best fitting model of nucleotide substitution determined by IQTREE. For the multilocus dataset we used the partitioning scheme and model of nucleotide substitution estimated by Shreve et al.^56^.

Principal component analyses were conducted on the *COI* dataset in R 3.6.2^69^ using the adegenet 2.1.2^70^ and ade4^71^ packages.

Worth mentioning, searching the BOLD Identification System (IDS) for *COI* barcodes similar to the *S*. *saudiensis COI* haplotype of Rasool et al.^55^ returned a species-level identification entry of 100% similarity identified as *Solenopsis* sp. HI01 (sample ID: PKSP5221; deposited in: Harvard Museum of Comparative Zoology (MCZ); sequence ID: ASPNA1425-10.COI-5P; BIN ID: BOLD:AAN0050) collected from Hawaii (20°56′09.6″N 156°30′50.4″W) during 2010 and back to BOLD Jul-2011 historical records (https://v3.boldsystems.org/index.php/IDS_OpenIdEngine?historical=Jul-2011). This observation outlines the utility of the BLAST search tool in the BOLD identification engine and GenBank in fast and appropriate species-level assignment, in case of presence of similar data, and highlights how audit effort of molecular datasets affects interpretations.

### Scanning electron microscopy

The mounted specimens were coated with platinum and imaged using a scanning electron microscope, model JSM-6380 LA, located at the College of Science, King Saud University, at a resolution 3.0 nm (30KV, WD8 mm, SEI), accelerating voltage 0.5–30 kV, and a magnification of 85 ×–400 ×.

## Results

### Molecular phylogenetic analysis

**Figure 1:**
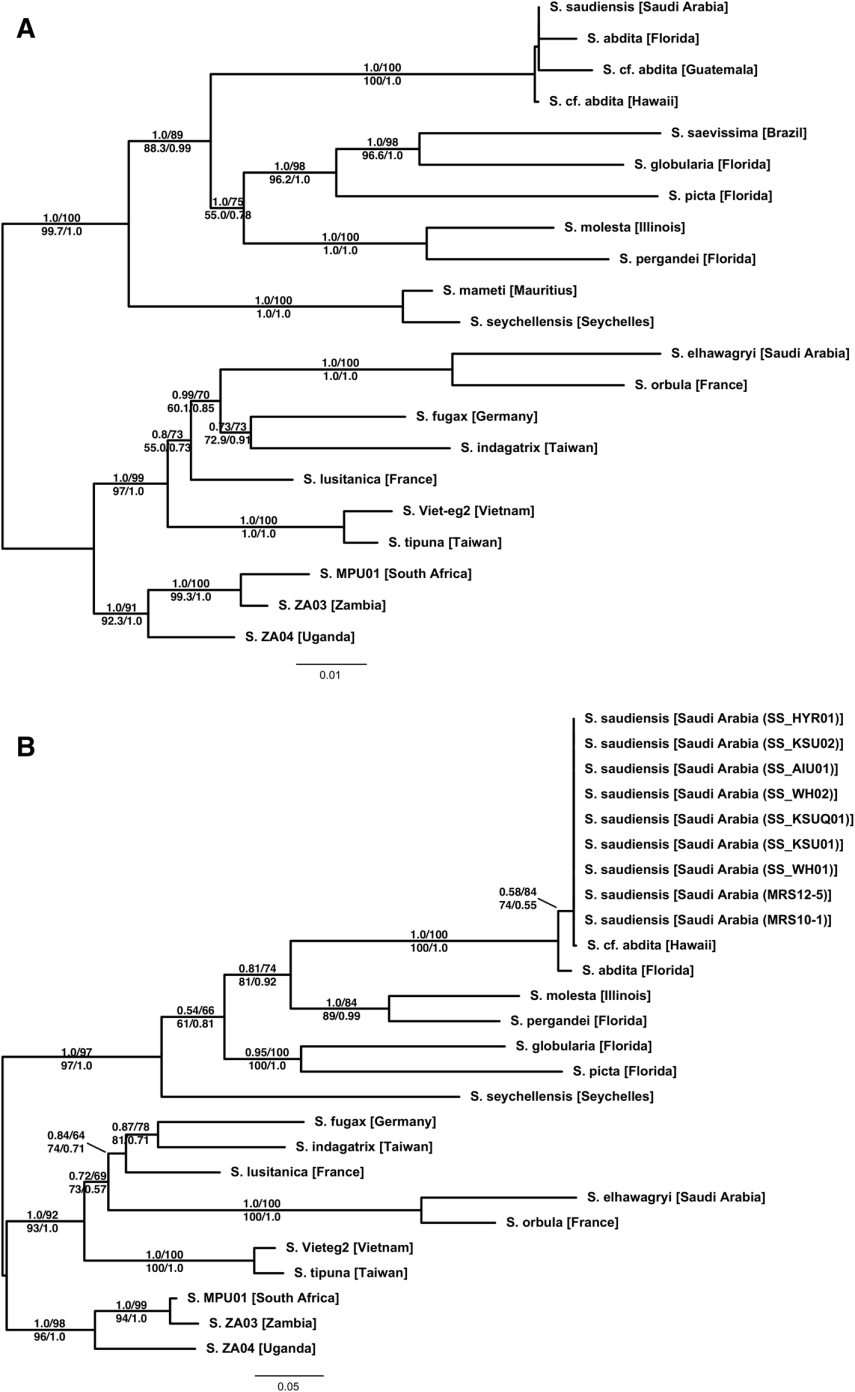
Phylogenetic trees of the multilocus (**A**) and the (**B**) mitochondrial *COI* datasets estimated using maximum likelihood (ML). The Bayesian phylogenies are fully compatible with the ML trees. Branch support is derived from ML and Bayesian posterior probabilities (above branch: posterior probability/ultra-fast bootstrap; below branch: SH-aLRT/aBayes).

Our molecular phylogenetic analyses of the multilocus dataset based on Bayesian and maximum likelihood (ML) methods (Fig. 1A) show clear and well-supported biogeographic patterns. The African, Eurasian, Nearctic, and Malagasy samples each fall into clades. The only exception is *S. saudiensis*, which forms a strongly supported (PP = 100, BS = 100, SH-aLRT = 1.0, aBayes = 1.0) clade with *S. abdita* from Florida and the two *S. cf. abdita* samples from Guatemala and Hawaii. Each locus individually supports the same pattern (not shown). However, nodes within this clade are all very poorly supported (PP < 10, BS < 60, SH-aLRT = 0.0, aBayes < 0.4). The analyses of only the *COI* data (Fig. 1B), which includes the Rasool et al.^55^ data, confirm the overall biogeographic pattern recovered with the multilocus dataset. Importantly, it also shows that all Saudi Arabian *S. saudiensis* samples and the single Hawaiian *S. cf. abdita* sample all share an identical *COI* haplotype.

**Figure 2:**
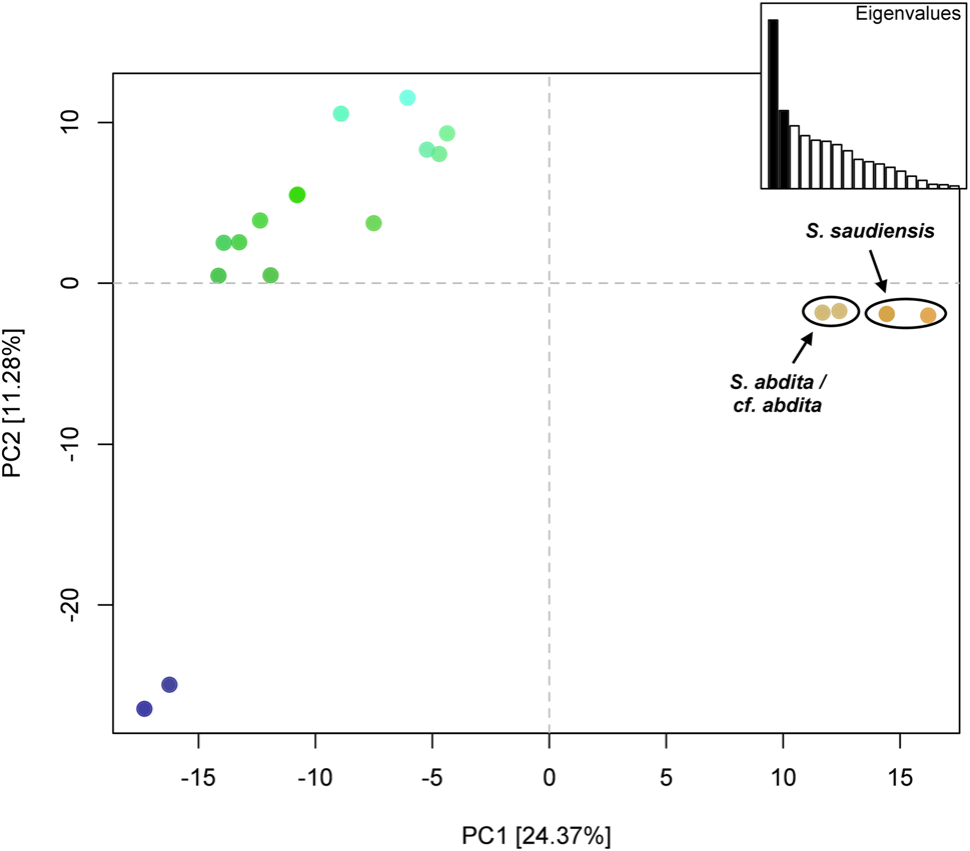
Principal component analysis (PCA) of *COI* dataset. The first two principal components are depicted, which together explain 35.65% of the variance. Points representing *S*. *abdita* and *S*. *saudiensis* haplotypes are labeled in brown and primarily differentiated by the first principal component. Colors are assigned by projecting the loadings of the first three principal components onto the RGB color channels.

The principal component analysis of the *COI* dataset shows three clusters (Fig. 2). The first principal component, which explains 24.37% of the variation and more than twice that of the second principal component, clearly separates the *S. saudiensis* and *S. abdita* samples from the other *Solenopsis* species. The *S. saudiensis* and *S. abdita* samples are poorly separated, and their differentiation is mainly derived by the first principal component.

According to the present work, *S. elhawagryi* is clearly associated with other Eurasian species and quite distinct from *S*. *saudiensis*, which falls out in a clade of Nearctic species. The molecular results are supported by our morphological analyses. The two species are easily separated based on the possession of the presence/absence of postpetiolar teeth and polymorphy/monomorphy of the worker castes. The former species has a postpetiole process in all castes and is polymorphic, whereas the latter lacks a postpetiole process and is clearly monomorphic.

### Morphological reassessments/new synonymy

**Figure 3:**
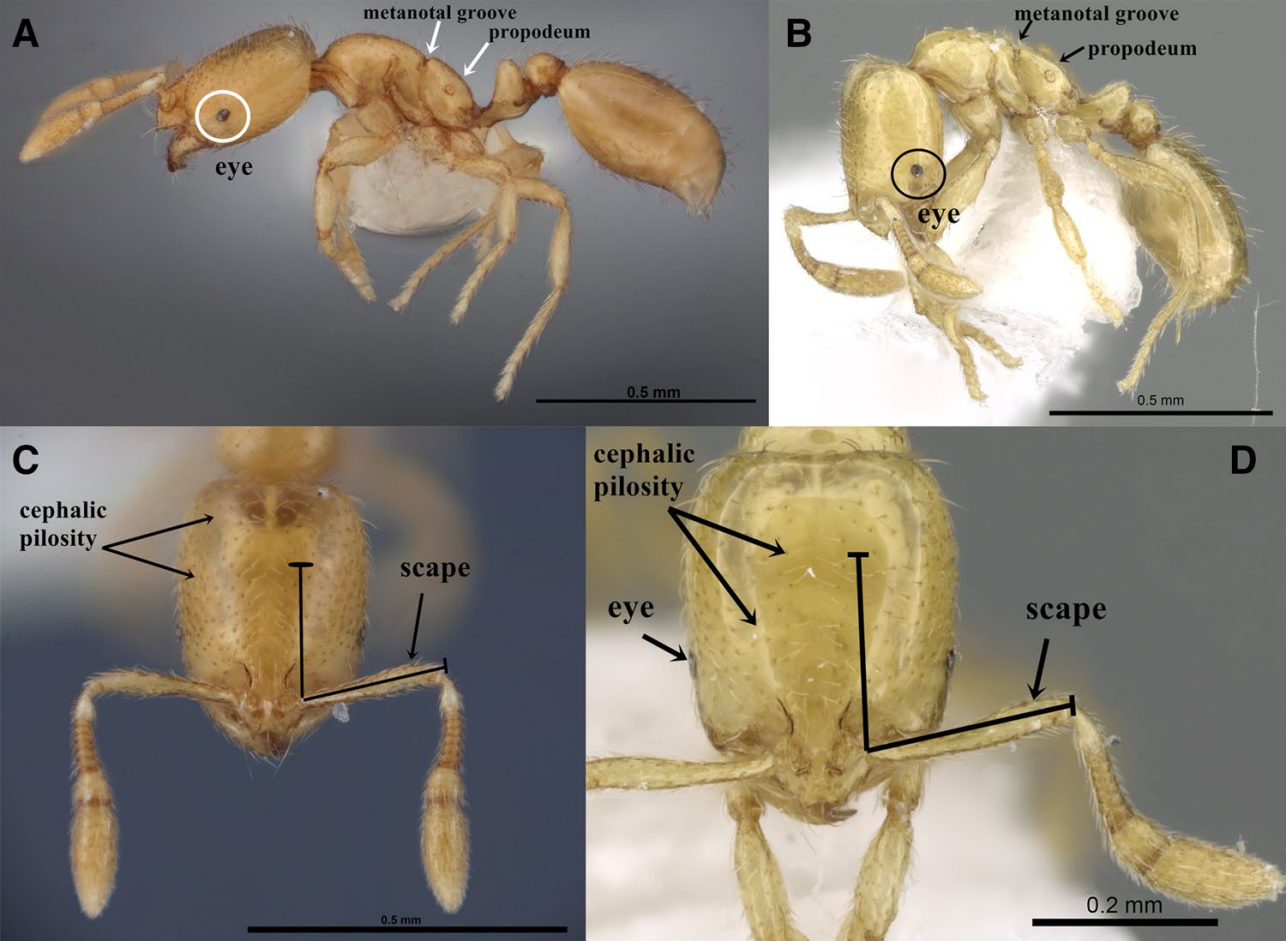
(**A**–**D**) Automontage images of *S. abdita* and *S. saudiensis*, (**A**, **C**) *S. abdita*, (**A**) body in profile, (**C**) head in full-face view, (FMNH-INS0000078522), Florida, (Photographer: Gracen Brilmyer); (**B**, **D**) paratype worker of *S. saudiensis*, (**B**) body in profile, (**D**) head in full-face view, (CASENT0249866), Saudi Arabia, (Photographer: Ryan Perry), from https://www.antweb.org/.

The genus *Solenopsis,* comprised of some of the smallest ants in the subfamily Myrmicinae, includes numerous minute species with minor workers less than 2 mm length. The material of *S. abdita* and *S. saudiensis* are ideally studied with high magnification microscopes, the Leica M205 C Stereomicroscope with a magnification zoom range of 20.5 × to examine and detect diagnostic characters that demonstrate clear morphological similarities between the two species. These similarities can be summarized in the following diagnosis (Fig. S1A–F, Fig. S2A–F) (Fig. 3): monomorphic species.

**Head.** Eyes minute with two to five ommatidia only seen with higher magnification, more frequently two; funicular segments 3–8 about twice as broad as long; anterior clypeal margin with a central pair of stout projecting teeth and a lateral pair of short, broad, basal, blunt teeth. **Mesosoma.** Dorsum of mesosoma smoothly curved, not flattened before and after the metanotal groove; metanotal groove acutely impressed in profile. **Postpetiole.** Postpetiole about 1.3 × broader than long in dorsal view; nearly hexagonal in profile with a distinctly convex dorsal surface. **Pilosity.** Relatively abundant and long hairs sparse on mesosoma, petiole, postpetiole, and gaster; more than 10 erect hairs on the dorsum of promesonotum; posterior tibial hairs mostly appressed. **Sculpture.** All body surfaces smooth and shining. **Color.** Uniform yellow or golden yellow, with coarse punctures present on the dorsum of the head.

The similarities include the body size and measurements of different body parts as follows: *S. abdita*: TL 1.02–1.20; HL 0.34–0.40; HW 0.28–0.34; EL 0.03; SL 0.22–0.24; ML 0.24; PW 0.07–0.09; PPL 0.072–0.084; PPW 0.10; Indices: CI 75–78; SI 58–67 (n = 18)^3,36^.

*S. saudiensis*: TL 1.2–1.3; HL 0.31–0.40; HW 0.28–0.31; EL 0.02; SL 0.21–0.27; ML 0.31–0.38; PL 0.10; PW 0.10; PPL 0.10; PPW 0.10–0.13; Indices: CI 75–87; SI 70–90; (n = 12)^28,29^. The previously mentioned morphological and molecular similarities of the two species support the synonymization of *S. saudiensis* with *S. abdita* syn. nov.

### Diagnostic and comparison with other *Solenopsis* species

Our molecular data reflects an apparent degree of similarity between *S. saudiensis* and *S. molesta*. The two species are similar in size and color, but the latter can be separated by the relatively larger eyes consisting of three to five ommatidia whereas *S. saudiensis* has distinctly smaller eyes with only two ommatidia. In addition, *S. molesta* has a head that is both longer and broader (HL 0.42–0.51, HW 0.36–0.43) than *S. saudiensis*.

Comparing the queen caste of *S*. *saudiensis* described by Sharaf et al.^72^ with the queen of *S*. *molesta* (Say, 1836) confirms that the two species are morphologically unrelated. The queen of *S*. *saudiensis* is uniform dark brown or black-brown, consistently smaller (HL 0.53–0.55, HW 0.46–0.50, EL 0.12–0.17), with a pointed petiole node profile and lacks a postpetiolar pair of teeth, whereas the queen of *S*. *molesta* is uniform yellow with darker mesosomal dorsum, distinctly larger (HL 0.72–0.84, HW 0.64–0.78, EL 0.24), a broad petiolar node rounded in profile, and postpetiole with a distinct subpetiolar pair of teeth.

Comparing *S. abdita*/*saudiensis* to *S. pergandei* (Forel, 1901), the three species are uniform yellow with eyes consisting of two ommatidia and present a distinct acute metanotal groove. However, *S*. *abdita*/*saudiensis* can be easily separated by the distinctly longer head when seen in full-face view (CI 75–87); abundant, short, subdecumbent or appressed body pilosity; and well-developed anterior central and lateral pairs of clypeal teeth. By contrast, *S. pergandei* has a nearly quadrate head (CI 89–93); profuse, suberect, and longer body pilosity; and a blunt central pair of anterior clypeal teeth while the lateral pair is absent.

The analysis of Rasool et al.^55^ shows a sister cluster of two unrelated species, *S. mameti* Donisthorpe, 1946 from Mauritius and *S. saevissima* (Smith, 1855) from Brazil. Morphologically, *S*. *abdita*/*saudiensis* and *S. mameti* are clearly distinct, as the former is uniformly yellow, with a shallow metanotal groove and less abundant, short, scattered body pilosity, whereas the latter is unicolorous dark brown with a deep metanotal groove and abundant, long body pilosity. *Solenopsis saevissima* is completely different from *S*. *abdita* / *saudiensis* and easily separated by numerous sets of characters including brown color, strong polymorphism in any nest series, profuse, stiff, and long body pilosity, conspicuously large eyes that contains about 12 ommatidia in the longest row, and an emarginated posterior margin of head seen in full-face view.

### *Solenopsis abdita*/*S*. *carolinensis*

Among the Nearctic species, *S. abdita* can be confused with *S*. *carolinensis* Forel, 1901 and Thompson^36^ was not able to present a practical differential diagnosis between the two species. However, Pacheco and Mackay^3^ successfully recognized the former species by the shorter scape, the broader petiole, and the appressed hairs on the tibiae whereas *S*. *carolinensis* has the tibiae with suberect hairs. In addition, the queen caste can be useful in the identification where the queen of *S. abdita* is dark brown and has smaller eyes, while the queen of *S*. *carolinensis* is yellow.

### Additional material examined

*Solenopsis abdita* (Fig. S1A,C, E; Fig. S2A,C, E; Fig. 3A, C): USA, Florida, Monroe Co., Key Largo, Hammock Botanical S.P., 25°10.524ʹN, 080°22.120ʹW, 10 m, 10.x.2010, (Corrie S. Moreau), (CSM1918), FMNH-INS 0000078522, 1 w, [FMNH]; USA, Florida, Osceola Nat. For., Baker Co., 10.07.1993, M. Deyrup, CASENT0104494, 1 w, (image examined).

*Solenopsis mameti*: MAURITIUS, 26.xii.1946, (R. Mamet), holotype w, (CASENT0102281), [BMNH].

*Solenopsis molesta*: USA, Virginia, (Pergande), 1015389, 1 w, CASENT0902336, [BMNH].

*Solenopsis saevissima*: BRAZIL: Syntype w, CASENT0902353, [BMNH]; syntype w, Blumenau, (Mme. V. Steiger), [NHMB].

*Solenopsis saudiensis* (Fig. S1B,D,F; Fig. S2 4B,D,F; Fig. 3B,D): SAUDI ARABIA: Riyadh, 24°43ʹN, 46°37ʹE, 9.VII.2009, 612 m (Mostafa R. Sharaf & Abdulrahman S. Aldawood), holotype w; two paratype workers with same data as the holotype, CASENT0217364; 117 paratype w, Riyadh, Wadi Hanifa, 24°39ʹN, 46°36ʹE, 15.I.2010, 633 m (Mostafa R. Sharaf & Abdulrahman S. Aldawood), CASENT0249866; 2 dealated q, Riyadh, King Saud University campus, 24°42.832′N, 46°37.534′E, 660 m, 04.iv.2014, (S. Salman), CASENT091433; Riyadh, King Saud University campus, 24.71383°N, 46.62557°E, 660 m, 02.ii.2014, (S. Salman) (2 w); Riyadh, King Saud University campus, 24.71383°N, 46.62557°E, 660 m, 06.ii.2014, (S. Salman) (3 w); Riyadh, Al Emam University, 24.81658 N, 46.71162E, 650 m, 08.ix.2014, (S. Salman) (2 w); Riyadh, King Saud University campus, 24.71383°N, 46.62557°E, 660 m, 10.iii.2014, (S. Salman) (3 w); Riyadh, Rhawdet Khorim, 25.383100°N, 47.278533°E, 559 m, 18.ii.2014, (Al Dhafer et al.) (3 w); Riyadh, Wadi Hanifa, 24.73507°N, 46.57518°E, 674 m, 18.ix.2014, (S. Salman) (2 w); Riyadh, Al Qawayiyah. R-Al Harmaliyah, 24.29773°N, 45.14577°E, 786 m, 20.iv.2015, (Al Dhafer et al.) (2 w); Riyadh, Azulfi, Rowdhat, Alsabalah, 26.36760°N, 44.98560°E, 671 m, 20.v.2015, (Al Dhafer et al.) (1 w); Riyadh, Rhawdet Khorim, 25.383100°N, 47.278533°E, 559 m, 26.v.2012, (Al Dhafer et al.) (2 w); Riyadh, Al Emam University, 24.817056°N, 46.701842°E, 657 m, 07.v.2014, (Mostafa R. Sharaf) (3 w) [KSMA].

## Discussion

The field study conducted by Rasool et al.^55^ in the Riyadh region did not turn up evidence of *Solenopsis* species other than *S. saudiensis*. However, the sampling methods and efforts deployed in their study are insufficient to conclude that other *Solenopsis* are absent, as many localities, habitats, and microhabitats in the province, which has a high diversity of natural and agricultural habitats, were left unexplored.

Our molecular results are clearly consistent with the Arabian revision of the *Solenopsis* fauna^29^ and the morphological traits used in species recognition. Based on molecular data as well as a morphological reevaluation of both *S. abdita* and *S. saudiensis*, our results indicate that *S. saudiensis*, described in 2011, represents a junior synonym of *S. abdita*. Thompson^36^ states that types of *S. abdita* were deposited at the Museum of Comparative Zoology (MCZ), the Florida State Arthropod Collection in Gainesville (FSCA), and the Natural History Museum of Los Angeles County (LACM), but extensive searches in these museums were unable to locate the type materials. The absence of *S*. *abdita* types has been observed before by Pacheco and Mackay^3^. In many *Solenopsis* species, however, the morphological distinction of species on the basis of the worker caste is arduous (e.g. *S. iheringi* Forel, 1908 and *S. bicolor* (Emery, 1906); *S. johnsoni* Pacheco et al., 2013 and *S. melina* Pacheco et al., 2013; and *S. azteca* Forel, 1893 and minor workers of *wasmannii*-group^3,11^), therefore, the study of the sexual castes including queens and males represent a useful addition for species delimitation^11^. Here, the comparison of the reproductive female caste of *S. saudiensis* described by Sharaf et al.^72^ with the original description of the queen of *S. abdita* (Thompson, 1989) revealed that most of their taxonomic characters match, including body size, sculpture, pilosity and reflected a straightforward synonymy. The few minor exceptions include body color, which is dark brown in the former species and reddish brown to almost black in the latter species. However, coloration in ants presents wide variation within and between populations^3,11^.

Ecological similarities are also found in the nesting habits of the two species, since both species were encountered nesting in palm logs (family Arecaceae)^3,36^*. Solenopsis saudiensis* has been collected in or near date palm plantations, *Phoenix dactylifera* L., on the Arabian Peninsula^28^ and *S. abdita* has been reported to be commonly found in rotten palm logs in the USA^36^. The nesting preference of *S. abdita* includes a broad range of habitats that are either moist or mesic niches in Florida including sandhill, swamp forest, grass tussocks of seasonal ponds, bases of pines in flatwoods, hammocks, rotten wood and palm logs^36,73^, or bases of date palm trees in the KSA^28^ where nests are built near the soil surface^73^.

These results also demonstrate the non-native status of the populations of *S. abdita* within KSA and represent the first known record of this species in the Old World. Introduced populations are also characterized by a reduced genetic pool as a consequence of a bottleneck effect following their introduction; which we observed in the form of populations from Saudi Arabia and Hawaii presenting identical *COI* sequences. The presence of this species in two regions outside its native range (the Arabian Peninsula and Hawaii), coupled with particular morphological and ecological traits such as small body size, polygyny^36^, lestobiotic lifestyle, and association with disturbed environments, supports the tramp status of this species^74^. Indeed, individuals of *S. abdita* in KSA were commonly encountered in date palm groves^28^ and highly disturbed urban habitats (one of the two type series was found nesting under a discarded carpet next to a human settlement^28^) but also in more natural habitats such as nature reserves (e.g. Rawdhat Khorim^75^). However, nature reserves are not necessarily disturbance-free and sometimes even the most pristine reserve can have exotic species along roads or buildings. Together, these results contrast with the conclusion of Rasool et al.^55^ of *S. abdita* being strongly associated with and specialized to colonize date palm groves following an adaptive process involving a large and strong gene pool.

**Figure 4:**
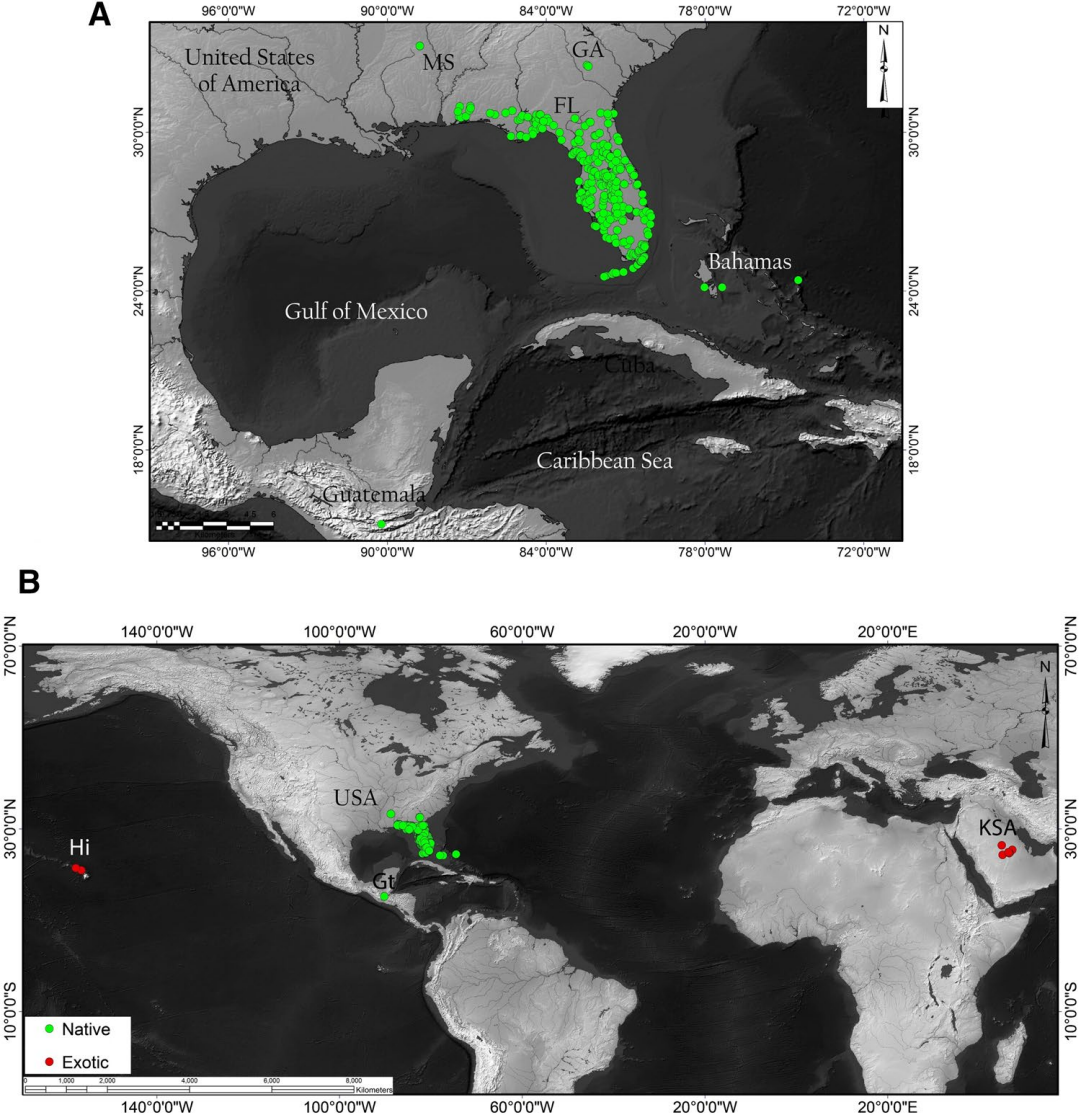
Worldwide distribution records of *S.* *abdita*. (**A**) Reported distribution in the United States (in green, native) with verified occurrences based mainly on http://antmaps.org^7,76^ and collection data from Dr. James Wetterer (Florida Atlantic University, USA). (**B**) Worldwide spread. Red dots indicate collection sites for samples used outside USA (exotic), including Guatemala, Hawaii, and Kingdom of Saudi Arabia (strict sense *S*. *saudiensis*). Maps were constructed using ArcGIS 10.3 software (Esri; Redlands, CA, United States).

While limited by the extent of the sampling used in our study, the results tend to indicate a New World origin for *S. abdita* potentially spanning the Nearctic and Neotropical realms. Given that the two samples from the Nearctic and the Neotropical regions (Florida and Guatemala, respectively) are genetically distinct and the species falls out in the New World clade, it seems likely that the native range is also somewhere in the New World (possibly circum-Caribbean). Currently, *S. abdita* is predominantly recorded from Florida and surrounding states (Fig. 4A; based on data from AntMaps^76^), which may entirely be an artifact of the geographic focus of the species keys used to identify thief ants.

If species of *Solenopsis* known as fire ants from the *geminata* (e.g. *S*. *aurea*, *S*. *geminata*, and *S*. *xyloni*) and *saevissima* (e.g. *S*. *invicta*, *S*. *richteri,* and *saevissima*) groups are notorious invaders in tropical to warm temperate climates regions^3,11,14,77^, this contrasts with the few successful introductions of the thief ants (previously referred as *Diplorhoptrum*) that have been recorded and their limited distribution within the introduced range (e.g. *S. globularia*, *S. papuana*, and potentially *S. terricola* to be confirmed as introductions in Florida). While an argument might be made for a potential candidate synonymy of these species with *S. abdita*, this possibility can be readily excluded by morphological examination. *Solenopsis globularia* (Smith, F., 1858) is easily distinguished by the greatly dilated/enlarged postpetiole seen in dorsal view, *S. papuana* Emery, 1900 has larger eyes that consist of three ommatidia plus a high profile of the propodeal dorsum, and *S. terricola* Menozzi, 1931 is a uniform dark brown species.

The identification of *S. abdita* as a new introduction within two distinct regions of the world [the Arabian Peninsula and Hawaii (Fig. 4B)] raises several questions. First, what is the extent of the native range of this species? And are populations from Guatemala part of the native or exotic range of this species? Second, due to the challenges of identifying of *S. abdita* and other thief ants, how many unidentified records of these species exist that potentially demonstrate a wider introduced range? Our study represents a case that could be expanded to more parts of the world to identify both *Solenopsis* specimens and other challenging ant taxa known to include major tramp species (e.g. *Cardiocondyla*^78^, *Pheidole*^79^, *Tetramorium*^80^).

Several of these taxa are widespread tramp species frequently involved in human‐mediated dispersal. Invasive and tramp species tend to have far-reaching geographical distributions and share life history traits including foraging behavior, nest structure, and queen number^9,16^.

Our phylogenetic and morphometric results indicate that invasive characteristics evolved within monomorphic *S.* *abdita*, such as its small size, lestobiotic lifeway, and phenotypic plasticity, could potentially confound taxonomists. Increased phylogenetic taxon sampling and improved species‐level taxonomy using ultrastructural tools will be necessary to explore the issue of invasive origins in further detail.

## Supplementary information

Supplementary Information.

Supplementary Table S1.

Supplementary Table S2.

## Data Availability

The specimens used in this study have been databased and the data are freely accessible on AntWeb (https://www.antweb.org). Main data needed to evaluate the conclusions in the paper are present in the paper. Additional data that support the findings of this study are available from the corresponding authors upon reasonable request.
