# Supplementary material for: Molecular phylogenetic analysis and morphological reassessments of thief ants identify a new potential case of biological invasions
Source: Sci Rep. 2020 Jul 21;10:12040. doi: 10.1038/s41598-020-69029-4 (PMC7374620; doi:10.1038/s41598-020-69029-4)
Supplement: Supplementary file 1 — Supplementary Information. [file 41598_2020_69029_MOESM1_ESM.pdf]

Supplementary information

Molecular phylogenetic analysis and morphological reassessments of thief ants identify a new potential case of biological invasions

Mostafa R. Sharaf[1*†], Dietrich Gotzek[2†], Benoit Guénard[3], Brian L. Fisher[4], Abdulrahman S. Aldawood[1], Hathal M. Al Dhafer[1] & Amr A. Mohamed[5†]

[1] Department of Plant Protection, College of Food and Agriculture Sciences, King Saud University, Riyadh, Kingdom of Saudi Arabia

[2] Department of Entomology, National Museum of Natural History, Smithsonian Institution, Washington, DC 20560, USA

[3] School of Biological Sciences, The University of Hong Kong, Hong Kong SAR

[4] California Academy of Sciences, San Francisco, CA 94118, USA

[5] Department of Entomology, Faculty of Science, Cairo University, Giza, PO Box 12613, Egypt

* corresponding author, e-mail: antsharaf@gmail.com & mosharaf@ksu.edu.sa

[†] These authors contributed equally

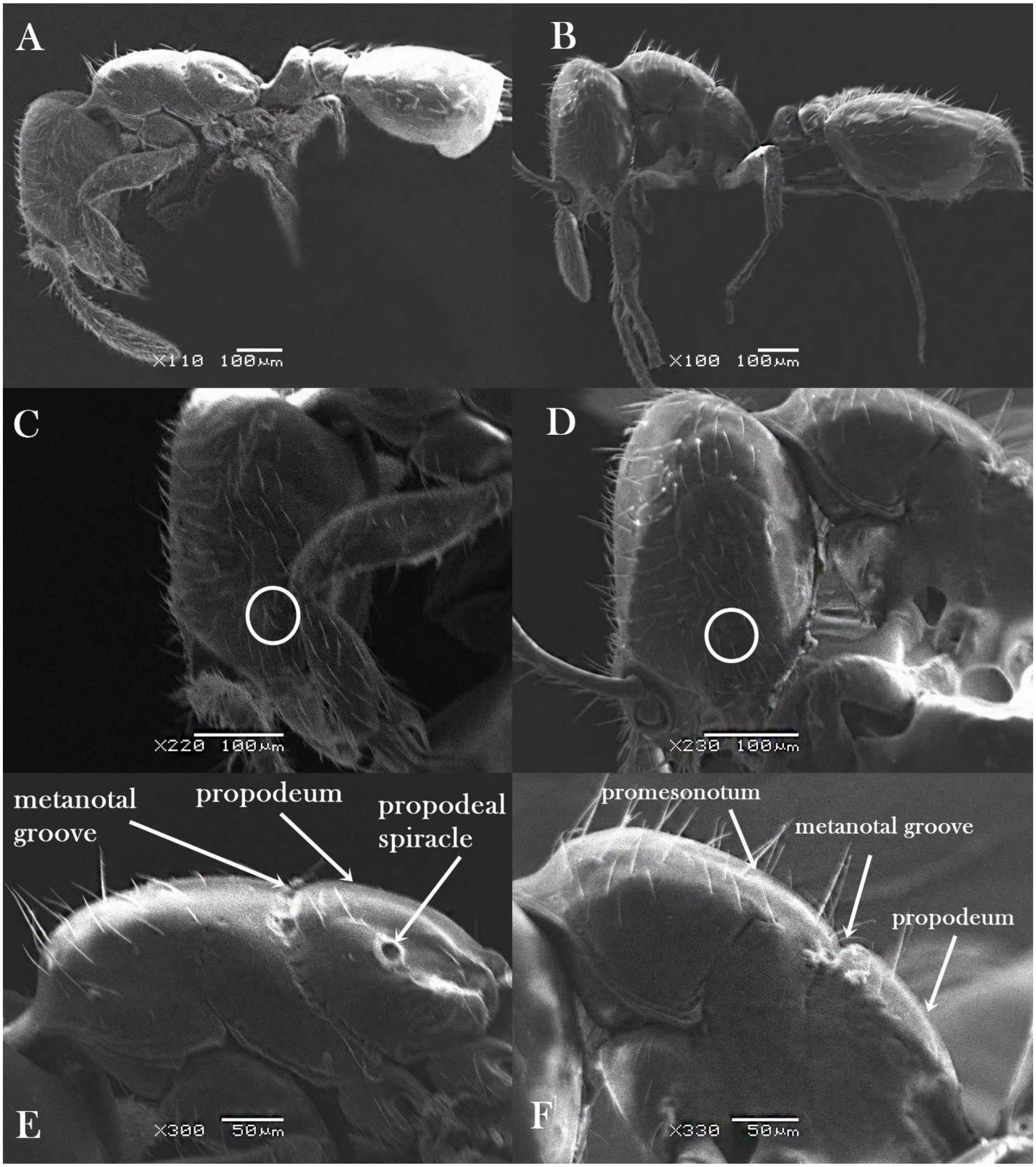

Fig. S1. A-F; **SEM images of a worker of *S. abdita* from Hawaii (left) and a paratype worker of *S. saudiensis* (right)**; A, B, body in profile, C, D, head in profile showing minute eyes; E, F, mesosoma in profile showing abundant pilosity, acute metanotal groove, and smoothly rounded propodeum.

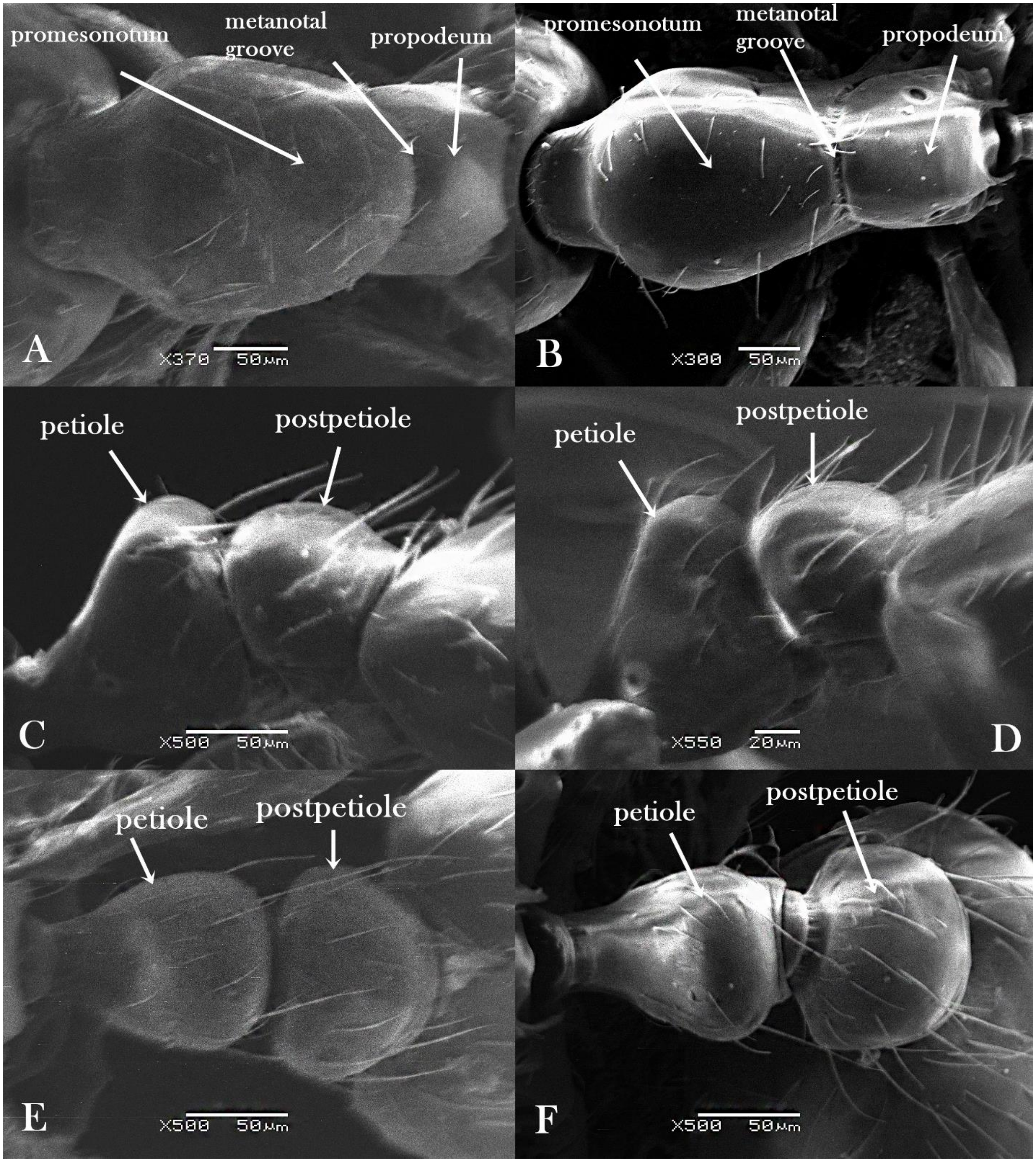

Fig. S2. A-F; **SEM images of a worker of *S. abdita* from Hawaii (left) and a paratype worker of *S. saudiensis* (right)**; A, B, mesosoma in dorsal view showing metanotal groove, and propodeum; C, D, petiole and postpetiole in profile showing higher petiolar node and abundant long pilosity; E, F, petiole and postpetiole in dorsal view.

Table S1. Information of physically accessible samples used in comparisons between *S*. *abdita* and *S*. *saudiensis*.

Table S2. GenBank accession numbers.
